# IL-17A in gastric carcinogenesis: good or bad?

**DOI:** 10.3389/fimmu.2024.1501293

**Published:** 2024-11-29

**Authors:** Weidong Li, Xiaodong Huang, Xiaowen Han, Jiayi Zhang, Lei Gao, Hao Chen

**Affiliations:** ^1^ Department of Surgical Oncology, Lanzhou University Second Hospital, Lanzhou, China; ^2^ Key Laboratory of Environmental Oncology of Gansu Province, Lanzhou University Second Hospital, Lanzhou, China

**Keywords:** gastric cancer, IL-17, tumor microenvironment, *Helicobacter pylori*, immunotherapy

## Abstract

Cytokines, which are important to the tumor microenvironment (TME), play critical roles in tumor development, metastasis, and immune responses. Interleukin-17(IL-17) has emerged as a key biomarker in many malignancies; however, its precise involvement in gastric cancer is less fully understood. Elevated levels of IL-17 have been observed in stomach diseases such as *Helicobacter pylori* infection and autoimmune gastritis, indicating that a sustained Th17 response may precede the development of gastric cancer. While IL-17 is related to inflammatory processes that may lead to cancer, its specific influence on gastric cancer development and therapy needs to be completely understood. Specifically, the release of IL-17A by diverse immune cells has been associated with both tumor development and inhibition in gastric cancer. It may impact tumor development through mechanisms such as boosting cell proliferation, inducing angiogenesis, and enabling immune cell recruitment or, conversely, suppressing tumor growth via the activation of anti-tumor immune responses. The dual role of IL-17 in cancer, along with its various effects depending on the TME and immune cell composition, highlights the complexity of its activity. Current research reveals that although IL-17 might serve as a target for immunotherapy, its therapeutic potential is hindered by its various activities. Some studies have shown that anti-IL-17 drugs may be helpful, especially when paired with immune checkpoint inhibitors, whereas others point to concerns about the validity of IL-17 in gastric cancer therapy. The lack of clinical trials and the heterogeneity of human tumors underscore the necessity for individualized treatment approaches. Further studies are needed to identify the specific mechanisms of IL-17 in gastric cancer and to design targeted therapeutics appropriately.

## Introduction

Cytokines play crucial roles in the development of the TME, affecting tumor growth, metastasis, and immune responses (1–4). Robust preclinical evidence supports therapeutic strategies aimed at enhancing the growth-inhibitory and immune-stimulatory effects of interferons and interleukins (including IL-2, IL-7, IL-12, and IL-15) or inhibiting the inflammatory and protumor effects of cytokines such as tumor necrosis factor(TNF), IL-1β, and IL-6 (5, 6). IL-17 has attracted significant interest as a new biomarker for tumor prognosis and immunotherapy in several malignancies, including colorectal, lung, and prostate cancers. Gastric cancer, the fifth most frequent cancer worldwide and the fifth major cause of cancer-related fatalities has minimal study on IL-17A, and its function in gastric cancer development and therapy remains contentious (7–10). Studies have revealed that IL-17 levels are higher in patients with autoimmune gastritis, gastrointestinal metaplasia, and atypical hyperplasia, indicating that a prolonged Th17 response may occur prior to the formation of gastric cancer. Persistent inflammatory states or immunological responses in the stomach area may generate physiological and morphological alterations in the gastric epithelium, increasing the likelihood of tumor transformation owing to diminished gastric acid and atrophy. Additionally, the increased inflammatory microenvironment might attract immune cells, changing the tumor’s immune environment and encouraging carcinogenesis and cancer progression (4). Bioinformatics analysis, immunohistochemistry, and sequencing data have revealed increased expression of IL-17 in gastric cancer tissues, although the specific link between IL-17 and gastric cancer remains uncertain. Therefore, this article seeks to evaluate the current research advances on the role of IL-17 in the development of gastric cancer and its applications in targeted and immunotherapy, summarizing its activities and effects to provide insights for individualized and precise treatment of gastric carcinoma.

## IL-17

The IL-17 family consists of six structurally similar cytokines, IL-17A-IL-17F, of which IL-17A has the highest percentage, and plays the most dominant function. IL-17A is produced mainly by Th17 cells, in addition to IL-17^+^CD8^+^ T cells(Tc17), γδT cells, natural killer cells (NK cells), neutrophil, and innate lymphoid-like cells can also secret IL-17A, which play an important role in inflammatory diseases and in the tumor environment (11, 12). Classical investigations have identified IL-17 as a major cytokine released by CD4^+^ T helper 17 (Th17) cells and CD8^+^ T cells in the inflammatory milieu that causes reactive oxygen species generation (13). IL-17 is highly expressed in a subset of patients with prostate, colorectal, gastric, breast, lung, and hepatocellular carcinomas, and some studies have shown that its expression level is positively correlated with tumor progression; however, some studies have shown that IL-17 is beneficial for human survival (14, 15). The IL-17 receptor family consists of five receptor subunits: IL-17RA, IL-17RB, IL-17RC, IL-17RD, and IL-17RE. IL-17RA was the first to be identified and contains a complicated structural motif consisting of similarly expressed fibroblast growth factor and IL-17r (SEFIR) and an extended sequence of SEFIR (SEFEX) (11, 16). NF-κb activator 1 (Act1) also has a SEFIR structural domain that interacts with IL-17RA/RC via the same SEFIR, which in turn activates all known IL-17-dependent signaling pathways (11). IL-17 promotes pro-inflammatory pathways, NF-κb, JNK, chemokines, and other pro-inflammatory cytokines (17).

**Figure 1:**
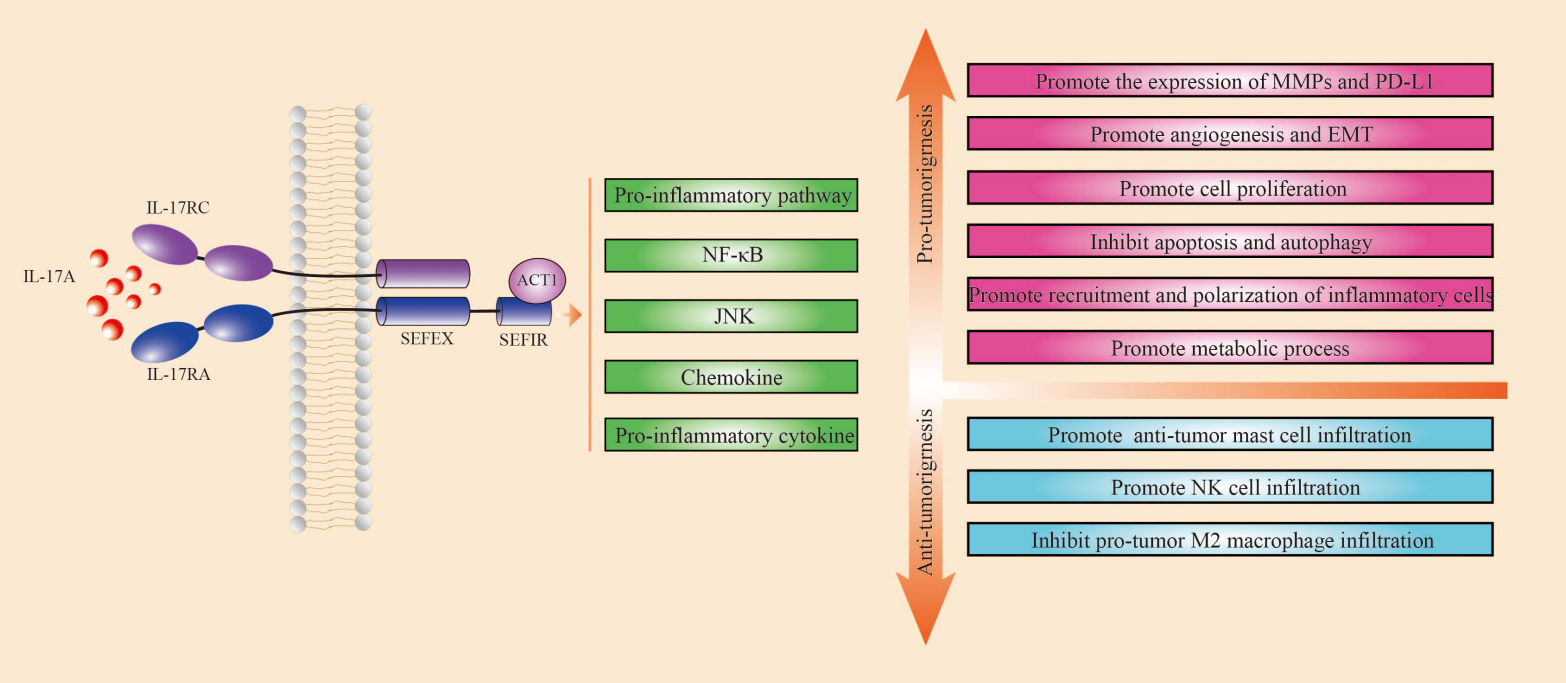
Mechanism of IL-17A in gastric cancer. Green squares indicate pathways activated by IL-17A, red squares indicate mechanisms that promote tumor progression, and blue squares indicate mechanisms that inhibit tumor progression. IL-17A acts on IL-17RA, which in turn activates IL-17A-dependent signaling pathways such as pro-inflammatory pathways, NF-κB and JNK, chemokines, and other pro-inflammatory cytokines through interactions with ACT1. IL-17A promotes tumor progression through several mechanisms, including enhancing cell proliferation, inhibiting apoptosis and autophagy, promoting the recruitment and polarization of inflammatory cells, facilitating metabolic processes, stimulating angiogenesis and epithelial-mesenchymal transition (EMT), and increasing the expression of matrix metalloproteinases (MMPs) and PD-L1. Other studies have shown that IL-17A may suppress tumor growth by boosting the infiltration of anti-tumor mast cells and natural killer cells while lowering the infiltration of pro-tumoral M2 macrophages.

Research has shown that IL-17A promotes tumor progression through various mechanisms, including enhancing cell proliferation, inhibiting apoptosis and autophagy, recruiting and polarizing inflammatory cells, facilitating metabolic processes, stimulating angiogenesis and EMT, and increasing matrix metalloproteinase (MMP) and programmed cell death 1 ligand 1(PD-L1) expression (11, 18–20). However, other studies have shown that IL-17A may suppress tumor growth by increasing the infiltration of anti-tumor mast cells and natural killer cells while lowering the infiltration of pro-tumoral M2 macrophages (**Figure 1**). Single-nucleotide polymorphisms (SNPs) in the IL-17 gene are associated with an increased risk of gastric cancer in East Asian populations, while no such association has been found in Latin American gastric cancer patients, indicating that the specific effects of IL-17 may vary by ethnicity and region (21, 22). Transient and controlled IL-17 levels induce physiological responses that are important in host defense and tissue repair, but persistent IL-17 activity is linked with pathogenic responses that promote cancer and autoimmunity (23). The existing studies are presented in **Table 1** (12, 18, 24–37). **Table 1** shows the results of the last 10 years of studies on IL-17A in gastric cancer, and the table includes the types of studies, methods, cell sources, and main conclusions drawn from the studies. The existing studies suggest that IL-17A can promote or inhibit tumor progression through a variety of mechanisms (**Table 1**; **Figure 1**). IL-17A is currently studied via a variety of methods, including immunohistochemistry, real-time polymerase chain reaction, enzyme-linked immunosorbent assay, and single-cell RNA-sequencing. Evidence regarding IL-17A promotion of tumors comes from a variety of sources, whereas evidence of tumor suppression is mostly derived from immunohistochemistry, suggesting that the heterogeneity of IL-17A assay methods or tumors may have an impact on outcomes.

**Table 1:** Roles of the cytokine IL-17 in gastric cancer.

Cellular source	Type	Material	Method	Response to IL-17 exposure	Conclusion	References	Year	country or region
Th17 cell	CLINICAL	human tissue	IHC	Anti-tumorigenesis	Patients with high levels of intratumoral IL-17A cells and low levels of peritumoral IL-17A cells in TNM stages II/III are more likely to benefit from ACT. Elevated IL-17A mRNA expression and increased intratumoral IL-17A cell infiltration are associated with enhanced infiltration of anti-tumor mast cells and natural killer cells, as well as reduced infiltration of pro-tumor M2 macrophages.	Intratumoral IL-17-producing cell infiltration correlates with antitumor immune contexture and improved response to adjuvant chemotherapy in gastric cancer	2019	China
Th17 cell	CLINICAL	human tissue	IHC, RT-PCR	Pro-tumorigenesis	The expression levels of IL-6, IL-17, FoxP3, and TGF-β1 in cancerous tissues were significantly higher than those in the control group and were associated with clinical staging of gastric cancer.	Expression of Th17/Treg related molecules in gastric cancer tissues	2018	China
Th17 cell	CLINICAL	human tissue, peripheral blood	ELISA kit	Pro-tumorigenesis	IL-17 and RORγt expression levels were significantly elevated in gastric cancer tissues and peripheral blood mononuclear cells, particularly in metastatic patients, with increased plasma IL-17 levels; it is suggested that the expansion of Th17 cells may contribute to the occurrence and metastasis of gastric cancer.	Th17 cell expansion in gastric cancer may contribute to cancer development and metastasis	2014	China
Tc17, Th17	CLINICAL	human tissue, peripheral blood	scRNA-seq	Pro-tumorigenesis	IL-17 cells may promote tumor progression through IL-17, IL-22, and IL-26 signaling pathways; thus, targeting IL-17 cells and related signaling pathways may serve as a therapeutic strategy for gastric cancer.	scRNA-seq of the gastric tumor shows complex intercellular interaction with an alternative T-cell exhaustion trajectory	2022	China
Tc17	CLINICAL	YTN2 , YTN16	scRNA-Seq, flow cytometry		CD4^+^ T cells and γδ T cells in tumors produce IL-17. The IL-17 and neutrophil axis is indeed a reason for resistance to PD-1 therapy; combined blockade of these two signals successfully eradicated YTN16 tumors in a CD8^+^ T-cell-dependent manner.	Deep immunophenotyping at the single-cell level identifies a combination of anti-IL-17 and checkpoint blockade as an effective treatment in a preclinical model of data-guided personalized immunotherapy	2020	Japan
Neutrophil	PRECLINICAL	SGC-7901 , BGC-823, BALB/c nude mouse	qRT-PCR	Pro-tumorigenesis	Tumor-educated neutrophils activate the AKT and p38 pathways in mesenchymal stem cells by secreting inflammatory factors (including IL-17, IL-23, and TNF-α), facilitating their conversion into cancer-associated fibroblasts (CAFs), which promotes the proliferation, migration, and invasion of gastric cancer cells *in vitro* and accelerates their growth and metastasis *in vivo*.	Tumor-Educated Neutrophils Activate Mesenchymal Stem Cells to Promote Gastric Cancer Growth and Metastasis	2020	China
Neutrophil	PRECLINICAL	GES-1, MKN45MKN74	IHC, IF, ELISA kit	Pro-tumorigenesis	TANs secrete IL-17a, enhancing the migration, invasion, and EMT of gastric cancer cells, while activating the JAK2/STAT3 signaling pathway in these cells.	Tumor-associated neutrophils induce EMT by IL-17a to promote migration and invasion in gastric cancer cells	2019	China
Neutrophil	CLINICAL	SGC7901, BGC-823, AGS	IHC	Pro-tumorigenesis	IL-17^+^ neutrophils comprise a significant portion of the IL-17-producing cells in human gastric cancer. The pro-inflammatory IL-17 serves as a key mediator for CXC chemokines in recruiting neutrophils to the invasive edge, and a high level of infiltrating neutrophils at the invasive margin is positively correlated with angiogenesis progression in gastric cancer patients.	Interleukin-17-Producing Neutrophils Link Inflammatory Stimuli to Disease Progression by Promoting Angiogenesis in Gastric Cancer	2017	China
IL-17A+ cell	CLINICAL	tissue	IHC	Anti-tumorigenesis	Patients with high expression of CD155 and low expression of CD3, CD4, CD8, IL-17, IFN-γ, or CD19, as well as those with high expression of both CD155 and α-SMA, have a poor prognosis.	Clinical significance of CD155 expression and correlation with cellular components of TME in gastric adenocarcinoma	2023	China
Gastric cancer cell	PRECLINICAL	HGC27, MKN45	RT-PCR, WB, ELISA kit	Pro-tumorigenesis	Cytokines in the TME, particularly those secreted by cancer cells, are key drivers of the transformation of NFs into CAFs. IL-17 secreted by gastric cancer induces NFs to convert into ‘CAF-like’ cells, thereby remodeling the TME; these ‘CAF-like’ cells can then secrete IL-8, which further promotes the proliferation and invasion of gastric cancer.	Cytokine-driven positive feedback loop organizes fibroblast transformation and facilitates gastric cancer progression	2022	China
IL-17 protein	PRECLINICAL	N87 , Fu97 , AGS, MKN-45	–	Pro-tumorigenesis	IL-17 upregulates the expression of SLPI and counteracts the inhibitory effect of cisplatin on gastric cancer cells.	LCN2 Mediated by IL-17 Affects the Proliferation, Migration, Invasion, and Cell Cycle of Gastric Cancer Cells by Targeting SLPI	2020	China
IL-17 protein	PRECLINICAL	MGC-803	RT-PCR	Pro-tumorigenesis	IL-17 induces EMT in quiescent gastric CSCs, significantly enhancing their invasion, migration, and tumor formation capabilities while promoting the activation of the downstream phosphorylation signaling pathway of the transcription factor STAT3.	The promotion of the transformation of quiescent gastric cancer stem cells by IL-17 and the underlying mechanisms	2017	China
–	CLINICAL	human peripheral blood	ELISA kit	Pro-tumorigenesis	Compared to healthy individuals, patients with gastric cancer exhibit elevated serum levels of IL-17A, with overall survival (OS) significantly correlated with IL-2, IL-6, IFN-γ, IL-17A, NLR, and ECOG (all p < 0.05). The activity of IL-17A may contribute to resistance against anti-tumor immunity and play a role in treatment failure.	Serum cytokines and neutrophil-to-lymphocyte ratio as predictive biomarkers of benefit from PD-1 inhibitors in gastric cancer	2023	China
–	CLINICAL	human peripheral blood	ELISA kit	–	There is no significant difference in IL-17 levels between gastric cancer patients and healthy individuals. Early gastric cancer patients exhibit significantly higher average IL-17 levels, while late-stage gastric cancer patients have IL-17 concentrations comparable to those observed in healthy individuals.	Interleukins 17 and 23 in patients with gastric neoplasms	2016	Poland
–	Bibliometric Analysis	–	–	Anti-tumorigenesis	In early gastric cancer, low expression of IL-2 and IL-17 is associated with a favorable prognosis, while in advanced gastric cancer, the expression of these cytokines does not significantly impact survival rates. It is speculated that they may influence the prognosis of gastric cancer at the time of onset.	Bibliometric Analysis of γδ T Cells as Immune Regulators in Cancer Prognosis	2022	China
–	Two-sample Mendelian randomization analysis	–		Anti-tumorigenesis	The circulating levels of IL-17 are negatively correlated with the risk of gastric cancer.	Genetically Predicted Circulating Levels of Cytokines and the Risk of Cancer	2022	Europe

## Gastric carcinogenesis is preceded by a protracted Th17 response, and serous IL-17 concentration is a reflection of the local inflammatory response in the stomach

**Figure 2:**
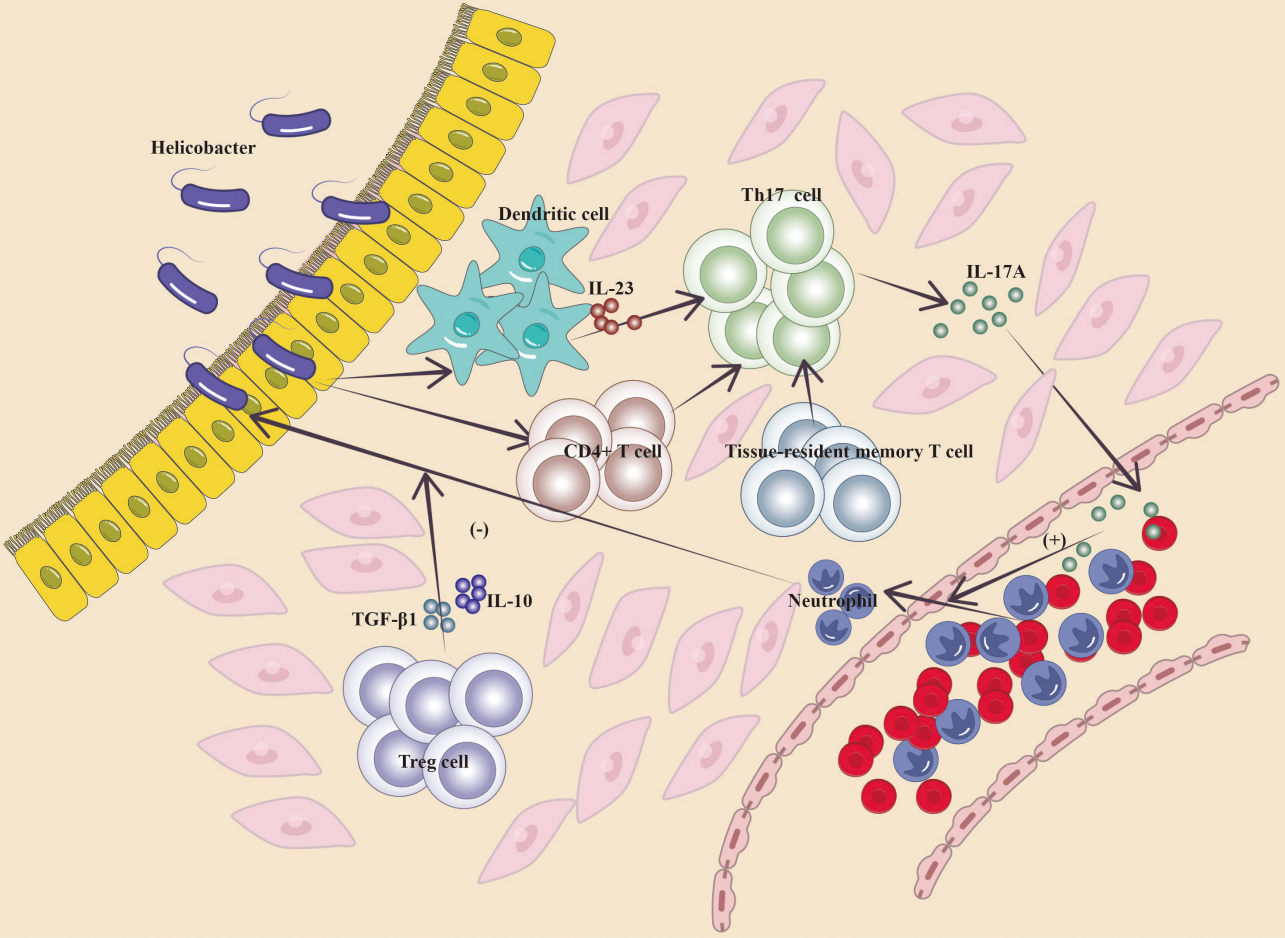
Mechanism of IL-17A in gastritis (Cytokines released by *H. pylori*-infected gastric stromal cells regulate the secretion of IL-23 by DC cells and monocytes, which promotes IL-17 expression and the developmental capacity of Th17 cells, and similarly, immunity to bacteria induces the transformation of naïve T cells, tissue-resident T cells in gastric tissues, to Th17 cells, which cause them to secrete IL-17A, which promotes the recruitment of inflammatory cells; ultimately, Treg cells exert an inhibitory effect on the Th17-induced inflammatory response by secreting the regulatory cytokines TGF-β1 and IL-10, and the ratio between Th17 cell and Treg responses determines the outcome of the local inflammatory response in the stomach).

The function of IL-17 in gastrointestinal chronic inflammation has been well researched, and IL-17 cytokine-producing lymphocytes defend barrier tissues from harmful microbes but are also key effectors of autoimmune disorders and inflammation (10, 38). Elevated levels of IL-17A have been detected in patients with autoimmune gastritis, gastrointestinal metaplasia, and atypical hyperplasia, indicating that a protracted Th17 response may occur before the development of gastric cancer (39, 40). Bella et al. reported increased blood levels of IL-17A, IL-17F, IL-21, and IL-17E in patients with autoimmune gastritis compared with normal individuals (40). In populations infected with *Helicobacter pylori*, serum IL-17A levels are considerably elevated in patients with gastrointestinal metaplasia and atypical hyperplasia compared with those with non-atrophic gastritis and normal individuals (39). Jenni Adamsson et al. reported an increased quantity of interferon (IFN)-γ^+^ and IL-17A^+^ cells among *H. pylori*-infected people, notably in those with *H. pylori*-induced stomach ulcers (10, 41). Cytokines secreted by stomach stromal cells infected with *H. pylori* may control the release of IL-23 by dendritic cells (DC) and monocytes, hence promoting IL-17 expression and the formation of Th17 cells (42, 43). Trefoil factor 1 can decrease the development of pro-inflammatory factors such as IL-17 by inhibiting the interaction between *H. pylori* and stomach stromal cells (44). The balance between Th17 and regulatory T (Treg) cells is critical for maintaining immunological homeostasis in the gastric mucosa since Treg cells exert inhibitory effects on Th17-induced inflammatory responses via the release of regulatory cytokines such as transforming growth factor (TGF)-β1 and IL-10 (45, 46). The Th17/Treg balance impacts the outcome of reactions to *H. pylori*; immune responses to bacteria can facilitate the transition of naïve T cells and tissue-resident T cells to Th17 cells in gastric tissue, potentially altering the ratio between Th17 and Treg responses, allowing IL-17-driven inflammation to surpass Treg responses and leading to bacterial clearance (45–48). In the context of *H. pylori* infection, IL-17 is required for bacterial clearance; nonetheless, its dual functional nature contributes to inflammation-induced illnesses (17). Mesenchymal stem cells (MSC) may reduce inflammatory responses and ameliorate the microenvironment (49). Injection of MSCs in animal models of gastric precancerous lesions may downregulate the concentration of IL-17 and interferon-γ in serum, indicating that IL-17 and IFN-γ may link with the intensity of inflammation in gastric lesions (50). Targeting different components, such as bacteria, host reactions, and cytokines, during chronic inflammation generated by *H. pylori* and subsequent carcinogenesis generates a complex network that reacts to inflammation and supports tissue healing. Epigenetic modifications related to cell proliferation, apoptosis, and tumor suppressor genes may occur, possibly leading to inflammation-related tumorigenesis (39, 51). IL-17A has been found to suppress the development of stomach precancerous lesions produced by chemical agents and *H. pylori*, resulting in concomitant decreases in stomach epithelial cell proliferation, oxidative stress, and the expression of gastric epithelial stem cell markers (52) (**Figure 2**).

However, an in-depth study of genetically related type I gastric neuroendocrine tumors in patients with autoimmune chronic atrophic gastritis suggested that the genetic origin of the mitochondrial alterations drives chronic inflammation of the stomach rather than the classical IL-17 secretion-mediated mechanism (13). Wojciech Błogowski et al. reported that the serum IL-17A concentration in patients diagnosed with other types of gastric tumors (gastrointestinal stromal tumors, gastric neuroendocrine tumors, and primary gastric lymphomas) was significantly lower than that in healthy individuals and patients with gastric adenocarcinomas and provided evidence that tumor progression is not only caused by IL-17 secretion (35).

Taken together, these findings suggest that a long-term Th17 response may precede the development of *H. pylori*-associated gastric cancer. The levels of IL-17 in serum, which reflects the degree of the local inflammatory response in the stomach, are largely released by Th17 cells, the expression of which is tightly tied to *H. pylori* infection. IL-17 levels may be lowered by reducing the interaction of *H. pylori* with stomach cancer epithelial cells. The eradication of *H. pylori* to avoid stomach cancer is of clinical importance. Subsequent clinical investigations of IL-17 should consider the presence or absence of *H. pylori* infection in patients. The pro-inflammatory features of IL-17 are crucial to its host-protective potential, although uncontrolled IL-17 signaling has been linked with immunopathology, autoimmune illness, and cancer development. However, the evolutionary pathway of gastric lesions due to genetic alterations, other inflammatory pathways, and autoimmune mechanisms cannot be excluded.

## IL-17A in gastric cancer tissues has a role in modifying the TME, with its function depending on the cellular origin

**Figure 3:**
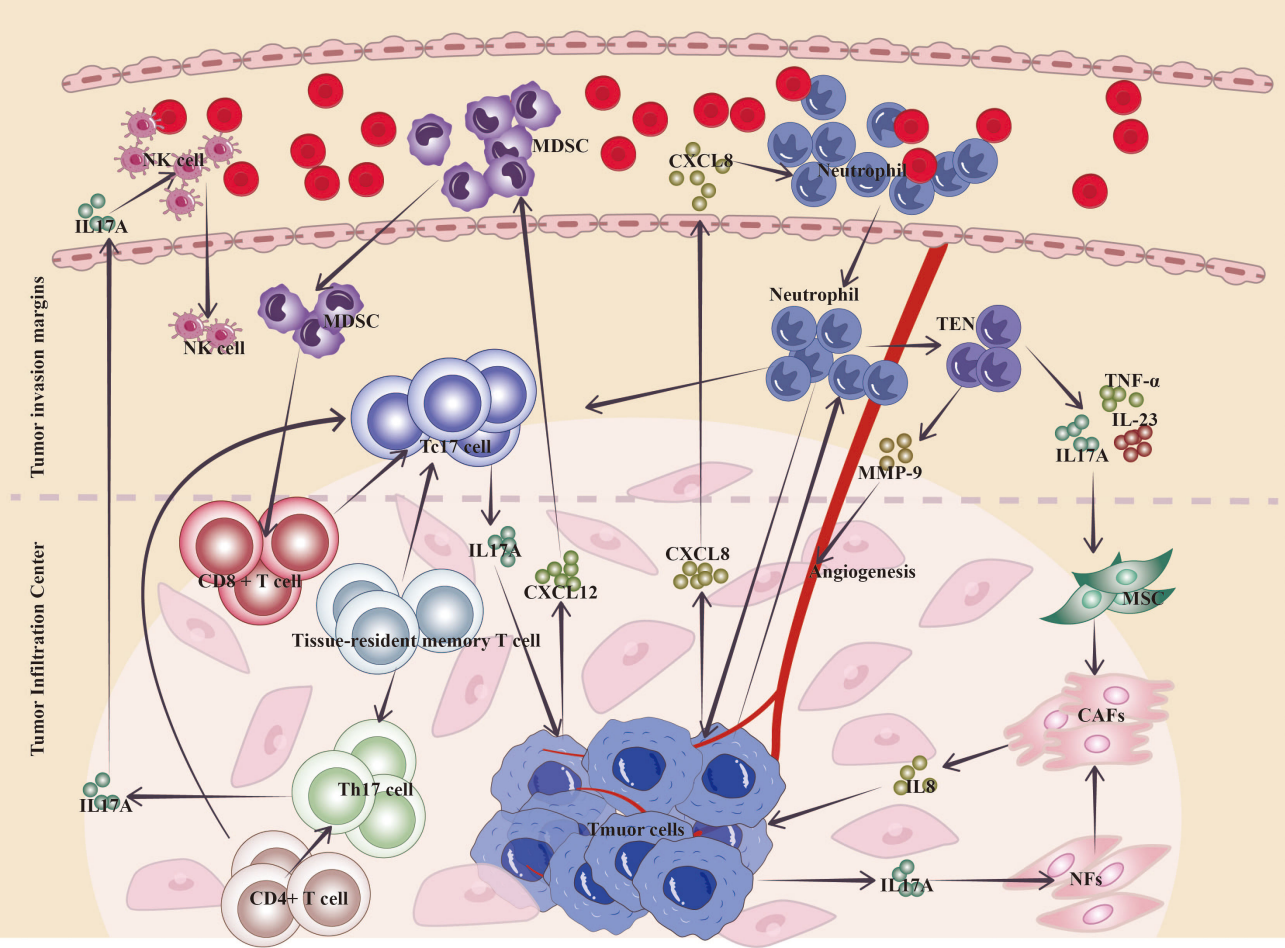
Mechanism of action of IL-17A in the gastric cancer TME. Neutrophils are widely distributed at the invasive margins of gastric cancer, and L17^+^ neutrophils migrate into gastric cancer through more neutrophils that can be induced by the production of chemokines (CXCL8, etc.) by cancer cells; tumor-educated neutrophils can secrete inflammatory factors, such as IL-17A, to activate the conversion of MSCs to CAFs, and tumor cells can also directly secrete IL-17 to induce the conversion of NFs to CAF transformation, while CAFs can secrete IL-8, etc., to promote gastric cancer proliferation and invasion; and neutrophils can change the TME toward tumor promotion by secreting MMP-9 to induce angiogenesis. Cancer-infiltrating CD8^+^ T cells and tissue-resident T cells may achieve depletion via Tc17 cells in the TME, and Tc17 cell-derived IL-17 drives cancer cells to release the chemokine CXCL12, which attracts MDSCs to suppress cytotoxic CD8^+^ T cells. Th17 cells in tumor tissues are located mainly at the center of tumor infiltration and are transformed from memory T cells and CD4^+^ T cells that accumulate during the inflammatory phase, and their infiltration correlates with increased survival and promotes the infiltration of cells such as anti-tumor natural killer cells. (TEN, tumor-educated neutrophils; MSC, mesenchymal stem cells; CAFs, cancer-associated fibroblasts; NFs, normal fibroblasts; NK cells, natural killer cells).

The continuing interaction between tumor cells and the TME (TME) is crucial in carcinogenesis, progression, metastasis, and response to treatment (53). The TME encompasses many cell types, including diverse immune cells, cancer-associated fibroblasts (CAFs), endothelial cells, and the extracellular matrix. The main infiltrating functional immune cells in gastric cancer include T cells, macrophages, NK cells, DCs, and myeloid-derived suppressor cells (MDSCs) (4). Previous investigations emphasized the discovery of IL-17 in patients with gastric cancer, although the involvement of IL-17A-positive cells in the cancer microenvironment has not been concluded (8). Most transcripts, including IL-17, are expressed at low levels (i.e., low abundance). Compared with whole-transcriptome analysis, low-abundance transcriptome sequencing can detect the vast majority of differentially expressed low-abundance transcripts, thereby identifying novel biomarkers, specific intracellular pathways, and gene targets that are aberrantly expressed in cancer tissues (54). Bizama et al. observed the overexpression of IL-17 in gastric cancer tissues through low-abundance transcriptome sequencing and concluded that IL-17 and IFN-γ are involved in the activation of T cells and B cells; moreover, IL-17 expression correlated with the activation of cytotoxic T-cell and NK cell pathways during *Helicobacter pylori* infection (55). Single-cell RNA sequencing of gastric cancer samples revealed the presence of Tc17 cells in the TME of most gastric malignancies. The IL-17A cluster corresponds to two separate traditional T-cell lineages (i.e., CD4^+^ and CD8^+^ T cells), but have the same gene expression patterns (12). The sources of IL-17 in the gastric cancer microenvironment are categorized as tumor cell-derived, Tc cell-derived, neutrophil-derived, or Th17 cell-derived, with different cell types infiltrating distinct regions (**Figure 3**).

Cytokines in the TME, especially those from cancer cells, are the main factors driving the transformation of normal fibroblasts (NFs) to CAFs; IL-17 secreted by gastric cancer cells can induce the transformation of NFs to CAFs and become “CAF-like” cells, which remodel the TME, and “CAF-like” cells can secrete IL-8, which promotes the proliferation of gastric cancer and the proliferation of the TME (32).

In addition to tumor cells, non-tumor cells within the TME can interact with MSCs via IL-17 to regulate their phenotype and function. Tumor-educated neutrophils (TENs) secrete inflammatory factors, including IL-17, IL-23, and TNF-α, which can activate the AKT and p38 pathways in MSCs, mediating their transformation into CAFs (28). The process promotes the proliferation, migration, and invasion of gastric cancer cells *in vitro* and accelerates their growth and metastasis *in vivo* (28). Neutrophils are broadly dispersed in the gastric tissues of gastric cancer patients, and their numbers dramatically increase, especially near the invasive borders of gastric cancer tissues, where neutrophil counts are notably greater than those in non-tumor tissue and the tumor core (29). Neutrophils inside the tumor tissue, particularly those generating IL-17, via secreting matrix metalloprotein 9 (MMP-9) promote angiogenesis and tumor progression (30). Research by Tuan-Jie Li et al. revealed the existence of large quantities of IL-17^+^ neutrophils in gastric cancer tissues; IL-17 promotes neutrophil migration into gastric cancer via chemokines generated by cancer cells, such as CXCL8 (30). Li et al. reported that tumor-associated neutrophils (TANs) are concentrated in gastric cancer, where they stimulate the JAK2/STAT3 signaling pathway in gastric cancer cells by secreting IL-17A, eventually promoting the migration, invasion, and EMT of gastric cancer cells (29).

Tumor cells release soluble factors that activate or modify monocytes in the TME to promote the polarization of Tc17 cells; IL-17 derived from Tc17 cells induces tumor cells to produce the chemokine CXCL12, thereby recruiting MDSCs to inhibit cytotoxic CD8^+^ T cells (56). Tumor-infiltrating CD8^+^ T cells and tissue-resident T cells may reach a state of exhaustion through the Tc17 trajectory inside the gastric cancer TME (12). Tc17 cells may stimulate tumor development via IL-17, IL-22, and IL-26, while non-cytotoxic Tc17 cells may become cytotoxic in the presence of IL-12, having a deadly impact on tumor cells (12, 57).

The involvement of Th17 cells in cancer formation may either promote or hinder tumor growth, depending on the type of cancer (45). Th17 cells may enhance EMT in colorectal and lung cancer cells, boosting tumor migration and metastatic spread (58, 59). Batf-dependent Th17 cells stimulate prostate cancer cell proliferation, block apoptosis, enhance inflammation, and accelerate tumor development via activating NF-κB signaling and the IL-23-IL-23R axis (60). Conversely, in chronic lymphocytic leukemia patients, an increase in Th17 cells is associated with improved prognostic markers and longer survival (61). In ovarian cancer and glioblastoma, Th17 cells are suggestive of increased survival rates, and many studies have demonstrated that generating Th17 immunity via DCs vaccines may increase the treatment results for glioblastoma and ovarian cancer (62, 63). In cervical squamous cell cancer, Th17 cells constitute a favorable response, but neutrophil-derived IL-17 is linked with poor prognosis (64, 65). Wang et al. assessed two distinct gastric cancer cohorts, The Cancer Genome Atlas cohort and Zhongshan Hospital cohort, and proposed that the primary IL-17A^+^ cells in the TME of gastric cancer are likely Th17 cells, which play a role in delaying tumor progression. An increase in tumor-infiltrating IL-17A^+^ cells correlates with greater infiltration of anti-tumor mast cells and natural killer (NK) cells, alongside reduced infiltration of pro-tumoral M2 macrophages; greater infiltration of Th17 cells in tumors is associated with improved survival (24). Targeting the STAT3 pathway in cancer cells may govern the growth of gastric tumors and impact immune cell polarization toward an anti-tumor Th17 population (66). Xue Liu et al. performed immunohistochemistry examinations of gastric cancer tissues and reported that patients with low expression of inflammatory markers, including IL-17, had inferior prognoses (31). Chen et al., through immunohistochemical testing and analysis of 192 patients with gastric adenocarcinoma, reported that low expression of IL-17 in tumor tissues is an independent predictor of poor prognosis, with patients expressing higher levels of IL-17 demonstrating significantly better five-year overall survival rates than those with lower levels of IL-17 (14). Bing Liu et al. conducted a bibliometric review and suggested that for early gastric cancer, low expression of IL-2 and IL-17 indicates a favorable prognosis, whereas, in advanced gastric cancer, the expression of these cytokines does not significantly affect survival rates, implying a potential influence on prognosis at the time of onset (36). However the expression of IL-17A may facilitate the differentiation of tissue-resident memory T (TRM) cells into CD8^+^ T cells and Th17 cells, leading to Trm cell exhaustion and subsequently impacting the efficacy of immunotherapy (67).

The inflammatory background of the TME impacts the capacity of cytokines to promote or inhibit tumor development. IL-17 may either promote or hinder tumor growth depending on its cellular origin. However, this capacity is not permanent; it may be altered by fluctuations in the types and numbers of immune cells and cytokines present in the TME. Numerous studies have demonstrated that IL-17A levels in the blood of cancer patients are associated with tumor stage. This correlation is attributed primarily to cytokines such as IL-17A secreted by tumor cells and Tc17 cells (primarily derived from neutrophils), which promote tumor progression by enhancing neutrophil infiltration at the invasive edges of tumors and facilitating the conversion of NFs and MSCs to CAFs. The role of Th17 cells located at the tumor infiltration center is controversial. While Th17 cells may increase the invasion of anti-tumor mast cells and macrophages, their activity may lead to T-cell fatigue inside the tumor tissue, leading to an immunosuppressive TME. The cytokine profile inside the TME may change the polarity and activity of IL-17^+^ cells in gastric cancer and further affect the infiltration of other immune cells.

## Relationship between IL-17 and gastric cancer treatment

There were over 968,000 new cases of stomach cancer in 2022 and close to 660,000 deaths, ranking the disease as fifth in terms of both incidence and mortality worldwide (7). The overall survival rate for stomach cancer patients, which is based on established treatment techniques, remains poor, with a five-year survival rate of approximately 25% across all stages. For patients diagnosed with advanced metastatic gastric cancer, the five-year survival rate is still less than 5% (9, 68). There is a compelling need to discover more effective immunotherapy-driven therapeutic options. Immunotherapy has proven successful in treating advanced gastric cancer; however, its advantages are confined to a subgroup of individuals. Immunological cells, together with the chemokines and cytokines they release, play a critical role in the immunological response to gastric cancer and considerably impact the effectiveness of immunotherapy (34, 69). Immunotherapy may induce the release of numerous cytokines, including IL-17, TNF-β, IL-1β, IL-2R, IL-6, IL-8, and IL-10, in advanced gastric cancer patients (70). Single-cell sequencing studies by Sun K et al. revealed that IL-17^+^ cells and the mechanisms mediating communication between IL-17^+^ cells and tumor cells offer viable therapeutic targets for treating IL-17-positive gastric cancer (12). The success of checkpoint inhibitors in cancer therapy is associated with the infiltration of TRM cells. The expression of IL-17A may facilitate the differentiation of TRM cells into CD8^+^ T cells and Th17 cells, leading to TRM cell exhaustion and subsequently impacting the efficacy of immunotherapy (67). In gastric cancer, there is a positive connection between IL-17A mRNA expression and the immune suppressive markers CD274, CTLA4, and LAG3 (24). Patients with greater IL-17A mRNA expression are likely to benefit more from inhibitors targeting CD274, CTLA4, and LAG3 (24). The targeted medication apatinib has been demonstrated to lower the expression of IL-17 in the serum of gastric cancer patients (71). Koji Nagaoka et al. demonstrated that blocking IL-17 with anti-IL-17 antibodies inhibited the growth of YTN16 tumors in subcutaneously implanted C57BL/6 mice. The combined blockade of IL-17 and PD-1 effectively eradicated YTN16 tumors in these mice, suggesting that the IL-17-neutrophil axis may contribute to resistance to PD-1 therapy (27). Fang Luan et al. evaluated patients with solid tumors, including gastric cancer, and reported that IL-17A levels rose in patients who benefitted from immunotherapy, but no such changes were identified in those who did not obtain clinical benefit from treatment (72). According to Wang et al., increased numbers of IL-17A cells inside tumors in patients with TNM stage II/III disease may correspond with better advantages from neoadjuvant treatment (24).

Current treatment techniques targeting IL-17A have focused mostly on patients with psoriasis and ankylosing spondylitis, whereas its application in cancer is largely restricted to preclinical animal trials. In follow-up research assessing the effects of the anti-IL-17A monoclonal antibody secukinumab on patients with psoriasis, psoriatic arthritis, and ankylosing spondylitis, it was observed that inhibiting IL-17A did not increase the incidence of malignancies in these individuals (73). In a study linked to alcoholic liver cancer, pharmacological inhibition of IL-17A/Th-17 cells via anti-IL-12/IL-23 antibodies was demonstrated to prevent the development of hepatocellular carcinoma (HCC) in alcohol-consuming mice (74). Additionally, therapy with antibodies that inhibit IL-17, IL-17RA, and IL-5 in Vk*MYC mice resulted in decreased accumulation of Th17 cells and eosinophils in the bone marrow, consequently slowing the development of multiple myeloma (75). Furthermore, the inhibition of IL-17A has been found to increase the tumor response to anti-PD-1 immunotherapy in microsatellite-stable colorectal cancer (76). However, there was a study indicated that blocking intestinal IL-17–IL-17RA signaling may reverse systemic anticancer effects (77).

Currently, relatively few studies have investigated the use of IL-17 in gastric cancer therapy, and no clinical trials have been conducted. Only one preclinical investigation demonstrated that blocking IL-17 in a mouse model of gastric cancer might reduce tumor development. The heterogeneity identified inside tumors, across malignancies, and the variable immune response characteristics among individuals hamper the development and translational implementation of these medicines (78). Gastric cancer demonstrates high intratumor, interpatient, and subtype heterogeneity (79). For example, there is significant diversity in the incidence of RAS/PI3K mutations among ERBB2-positive cohorts, which has major consequences for therapy selection and effectiveness among individuals (80). Considering the dual function of IL-17 in malignancies, only targeting IL-17 does not appear to be a smart approach, and a customized examination of patients is needed. The link between IL-17 and immunological checkpoints, such as CD274, and the impact of combining IL-17 with immune checkpoint inhibitors (e.g. PDL-1/L1), may be further investigated. Considering that it is difficult for anti-IL-17 drugs to be used for gastric cancer treatment, some studies have noted that apatinib can attenuate the expression of IL-17 in the serum of patients with gastric cancer, or it may be possible to explore whether there is a difference in the therapeutic effect of apatinib on patients with high or low expression of IL-17.

## Conclusion

The treatment landscape of gastric cancer has evolved significantly in the past few years, with the emergence of new immunotherapy and targeted therapies for patients at various stages of the disease (81). The inflammatory response in the TME for cancer therapy is a hot topic of current research, which represents a breakthrough in the existing therapeutic paradigm (8). A review of previous studies revealed that gastric cancer may be preceded by a long-term Th17 response, during which IL-17 is mainly secreted by Th17 cells, and the amount of IL-17 in the serum reflects the degree of the local inflammatory response in the stomach and is a marker of the persistent state of inflammation. The expression of IL-17 is closely associated with *Helicobacter pylori* infection. The shift in Th17/Treg balance to Th17 leads to the clearance of H. pylori but is also accompanied by a persistent local inflammatory response, which may ultimately lead to the development of inflammation-associated tumors. During the development of gastric cancer, it is mainly secreted by Th17 cells, Tc17 cells, neutrophils, and gastric cancer cells. IL-17 participates in the construction of the TME of gastric cancer and can play different roles according to the cellular source and the inflammatory background of the TME. Tc17 cells, neutrophils, and gastric cancer-derived IL-17A all exhibited tumor-promoting effects. The expression of IL-17A in Th17 cells may be associated with a better prognosis. Based on the current study, it can be concluded that whether IL-17A is good or bad in gastric cancer depends on the stage at which it is present and its cellular origin.

The inconsistent findings obtained in gastric cancer regarding the existence of pro- and anti-cancer activities of IL-17 may be attributed to the research population, H. pylori infection, the treatment received, the cellular source of IL-17, and the inflammatory background of the TME, and individualized and fine-tuned in-depth studies are needed to clarify the specific mechanism of its action in gastric cancer and to conduct further studies to develop IL-17-targeted anticancer strategies against IL-17. The current lack of clinical studies directly addressing IL-17 in gastric cancer treatment underscores the need for further investigations to delineate its effects more clearly. Ultimately, a deeper understanding of whether IL-17 acts more as a friend or foe in gastric cancer could lead to more effective, personalized therapeutic strategies tailored to the unique profiles of gastric cancer patients, integrating the distinct inflammatory contexts influencing disease progression.
